# Protean Kinematics: A Blended Model of VR Physics

**DOI:** 10.3389/fpsyg.2021.705170

**Published:** 2021-08-23

**Authors:** David C. Jeong, Steffie Sofia Yeonjoo Kim, Jackie Jingyi Xu, Lynn C. Miller

**Affiliations:** ^1^Department of Communication, Santa Clara University, Santa Clara, CA, United States; ^2^Annenberg School for Communication and Journalism, University of Southern California, Los Angeles, CA, United States

**Keywords:** VR/XR, locomotion, motion tracking and capture, neuroscience, game studies, kinematics

## Abstract

Avatar research largely focuses on the effects of the appearance and external characteristics of avatars, but may also warrant further consideration of the effects of avatar movement characteristics. With *Protean kinematics*, we offer an expansion the avatar-user appearances-based effects of the Proteus Effect to a systematic exploration into the role of movement in affecting social perceptions (about others) and idealized perceptions (about self). This work presents both a theoretical (typology) and methodological (physics-based measurement) approach to understanding the complex blend of physical inputs and virtual outputs that occur in the perceptual experience of VR, particularly in consideration of the collection of hippocampal (e.g., place cells, grid cells) and entorhinal neurons (e.g., speed cells) that fire topologically relative to physical movement in physical space. Offered is a novel method that distills the blend of physical and virtual kinematics to contribute to modern understandings of human-agent interaction and cognitive psychology.

## 1. Introduction

Social psychologists have long argued that human behavior is a function of both “persons” and “environment”, represented by the formula, *B* = *F*(*P, E*) (Lewin, 1936, 1943). Gibson's (1979) perceptual theory of affordances added that perceptual objects are understood in terms of the possible actions that the objects can engage perceivers, thus binding the existence of organisms to their environment. Following the above, the current work explores the intersection of the persons and the affordances of their environments across physical and virtual domains. We investigate this intersection particularly in the context of Virtual Reality (VR), examining the transfer from human users in physical environments to avatars in virtual environments [see Grabarczyk and Pokropski (2016) for a discussion of Gibsonian affordances and VR].

We anchor this work upon the conceptual framework of the Proteus Effect, which argues that physical cues of avatars direct behavioral and attitudinal changes among human users in accordance with the perceptions tied to the avatar appearances (Yee and Bailenson, 2007; Yee et al., 2009). The current work proposes to expand the logic of the Proteus Effect (Yee and Bailenson, 2007) from avatar-user appearances-based effects to a theory of movement-based effects^1^ vis–vis user and avatars in VR. We propose a systematic exploration into the role of movement in VR and virtual environments, both in terms of social perceptions (about others) and idealized perceptions (about self).

In the current work, we argue that VR represents a potential methodological tipping point for the social sciences. Whereas, computer-mediated communication focuses on a distinction between in-person and mediated communication, VR represents a blend of both. For instance, in-person communication examines physical cues, such as proxemics (Hall et al., 1968), while computer-mediated communication focuses on perceptions of such physical closeness, such as presence (Lee, 2004) and narrative transportation (Green and Brock, 2000). Recent research have applied such concepts in VR settings, such as in perceived realism (Shin et al., 2019), narrative immersion (Cummings et al., 2021) and conversational immersion (Oh et al., 2019).

VR, and more specifically the networked connectivity of social VR (e.g., VRChat, AltSpaceVR), represents a novel blend of the in-person (i.e., physical) and the computer-mediated (i.e., virtual) (Kruzan and Won, 2019; Oh et al., 2019). This is because social VR represents real-time virtual interactions between avatar representations of remote users, where in-person cues re-emerge as critical real-time factors. Further, the development and adoption of wireless 6 degrees of freedom (DOF) VR headsets affords greater flexibility in movements than what was possible with early VR headsets (e.g., tethered to PC, 3 DOF), thus enhancing the affordances of in-person communication.

Integration with social media functionality, online game communities, and wireless connectivity have all contributed to making VR an increasingly remote device^2^. Indeed, Kruzan and Won (2019) discuss the emerging blend between VR and social media on social VR platforms (e.g., Altspace VR, VRChat, Rec Room, Big Screen). It should be noted that the social media function of some of these platforms also blend with online game communities, or more specifically, online VR game communities. Whether approaching from a social media perspective or a game community perspective, it is important for media researchers to consider the increasingly socially networked nature of VR that embraces the interaction of remote VR users in virtual spaces. Critically, social VR platforms demonstrate that VR is more than a modality of representation (e.g., 360 VR), but a unique social experience with unique scripts (Schank and Abelson, 1975) and norms navigated between users. Altogether, it is increasingly important to consider the role of “cues" that occur at the intersection of physical inputs and corresponding virtual outputs.

The current work expands on the Proteus Effect by offering the following contributions: First, we introduce the concept of Social Kinematics, which provides the physics-based foundation to analyze and measure virtual movement, specifically in VR. Second, we explicate a typology of Protean Kinematics, which refers to movement-based effects at the intersection of physical affordances/actions and virtual affordances/actions. Finally, we present a computational model to compute physical to virtual movement, which researchers may ultimately use to more accurately and reliably measure the VR user actions and movements. In doing so, we aim to advance the replicability and transparency of measurement in the psychological and communication sciences, as noted by the open science movement in psychology (Ioannidis, 2012; Brandt et al., 2014; Nosek et al., 2015; Open Science Collaboration, 2015; Klein et al., 2018) and in communication (Benoit and Holbert, 2008; Bowman and Keene, 2018; McEwan et al., 2018; Keating and Totzkay, 2019; van Atteveldt et al., 2019; Dienlin et al., 2020; Lewis, 2020).

## 2. Related Work

We position this work at the intersection of traditional psychology and human-computer interaction. As such, we structure our related work to begin with relevant literature in traditional psychology. Next, we discuss work in media psychology, namely anchoring upon the Proteus Effect. Finally, we discuss connections to relevant work in human-computer interaction, which sets the stage for the introduction of our conceptual typology of Protean Kinematics.

### 2.1. Proteus Effect

The Proteus Effect (Yee and Bailenson, 2007) is arguably the leading media psychology theory on avatars as representations of self [see Praetorius and Görlich (2020) for a review]. According to the Proteus Effect, identity cues among user avatars may direct behavioral and attitudinal changes (Yee and Bailenson, 2007; Yee et al., 2009). Since its inception, the Proteus Effect has been explored in a wide range of contexts, such as dating (Yee et al., 2009), pedagogy (Ratan and Dawson, 2016), consumer choices (Ahn and Bailenson, 2011), public speaking anxiety (Aymerich-Franch et al., 2014), embodiment of elderly bodies (Beaudoin et al., 2020). One of the notable areas of application of the Proteus Effect has been in cross-gendered embodiment of avatars (Slater, 2009), such as in the context of gendered avatar customization and pedagogical stereotype threat (Lee et al., 2014; Ratan and Sah, 2015), stereotypically gendered behaviors (Sherrick et al., 2014), and sexualized self-objectification (Vandenbosch et al., 2017). A recent meta-analysis of 46 research studies on the Proteus Effect found a small-but-approaching-medium effect size (0.22 0.26), which is notably large relative to comparable meta-analyses on digital media effects (Ratan et al., 2020).

### 2.2. Real-Virtual Consistency

The question of avatars—not unlike digital media effects at large—starts and ends with a question of real to virtual consistency (Williams, 2010). That is, does the virtual avatar accurately represent the actual real-world self? Bente et al. (2001) demonstrate the overlap between real-world experiences and virtual representations of those experiences by finding only marginal differences in socio-emotional impressions between dyadic interactions and virtual animations of the same interactions. Beyond impressions, virtual environments afford opportunities to test for correlations between real-world decisions and virtual versions of those decisions, otherwise known as *virtual validity* (Godoy et al., 2008; Smith et al., 2018; Miller et al., 2019a,b,c). In addition to behavioral decisions, avatars may further influence behavioral change, such as those sought by health interventions. For instance, interventions may utilize avatars that represent a virtual future self that is older and wiser in order to influence (healthier) behavior and decisions (Christensen et al., 2013), akin to a self-fulfilling Bandura-esque process (Bandura, 2001).

Returning to our discussion of the Proteus Effect, it is thought that individuals infer their own attitudes and beliefs by taking on a third person perspective of oneself (Bem, 1972). Accordingly, it would seem to follow that the Proteus Effect would be best optimized for third person avatars. That said, the original Proteus Effect study (Yee and Bailenson, 2007) utilized first-person avatars based on the idea that first-person avatars elicited a stronger Proteus Effect than third-person avatars (Yee et al., 2009). Others have explored a hybrid first-person/third-person perspective, affording a combined benefit of first-person embodiment and third-person avatar appearance cues (Ratan and Sah, 2015).

### 2.3. Psychology of VR

The effectiveness of first-person perspectives may be attributed to embodiment (Barsalou, 2009), particularly in game avatars (Fox and Ahn, 2013; Ratan, 2013; Nowak and Fox, 2018). The merits of first person avatars in traditional games (e.g., Proteus Effect, embodiment, self-presence) translate conveniently in VR. Both take place in a first-person perspective where users' avatars are not readily viewable by the user. Certainly, a hypothetical user may meticulously craft a particular appearance of an avatar, but avatars in VR are generally used as cues for other users that interact with the given user.

Many have identified embodiment, such as embodied simulations (Riva et al., 2007, 2019; Riva and Gaudio, 2018), as one of the key conceptual phenomena underlying social presence (Lee, 2004) of avatar representations of users in virtual environments (Ratan and Sah, 2015; Ratan and Dawson, 2016; Ratan et al., 2020). VR extends this line of inquiry both literally and conceptually, as “cyborg prosthetics” (Biocca, 1997) present a paradoxical combination of the a) sensory and b) mental experience of agents, objects, and environments (Benthall and Polhemus, 1975).

That said, VR differs from traditional game environments in two prominent ways, both of which operate under the assumption of a first-person perspective. First, VR relies on “body illusions” that have roots in phenomena such as the “rubber hand illusion” (Botvinick and Cohen, 1998; Slater, 2009). Such body illusions have a powerful impact on a variety of psychological outcomes (Gonzalez-Franco and Lanier, 2017), such as effects on human perception (Cummings and Bailenson, 2016). This effect can be seen with “switched” hand controls from real to virtual hands (Bailey et al., 2016). Won et al. (2015) “playfully” pushed the envelope of such “homuncular flexibility” of somatic mappings by having a physical leg control a virtual arm, and vice versa (Won et al., 2015). More recently, Kocur et al. (2020a) leveraged new developments in hand tracking capacity across VR by exploring the impact of missing fingers in VR. Notably, representation of hands and limbs was observed to have no significant impact on various outcomes (e.g., body ownership, immersion, and emotional involvement) relative to VR representations excluding visual representations of limbs (Lugrin et al., 2018).

Second, unlike in traditional game environments, VR involves the added dimension of body kinematics, where users' actual body movements have a direct and corresponding virtual output on avatars' movements. That said, virtual avatar movements are certainly not constrained purely to the movement affordances of human bodies. For instance, a common movement in VR is the teleport movement, which allows individuals to point to and automatically move to a new location in a given space without having to physically “walk” to that location (Bozgeyikli et al., 2016).

## 3. Psychology of Movement

Consideration of movement in VR warrants a re-tracing of steps to the psychology of human movement. Indeed, we emphasize that the current work is an exploration of sensory perception (e.g., visual processing) and social perception (e.g., inference making). To address the sensory perception, we first discuss the role of hippocampal place cells (O'Keefe and Dostrovsky, 1971). Next, we turn our focus to the social perception of movement (Heider and Simmel, 1944).

### 3.1. Neuroscience and Movement

Human movement, and specifically locomotive movement, involves concurrent coordination of multiple brain systems. First, the sheer physical act of moving one's body requires activation of one's motor cortex. As one navigates from one point to another, visual inputs may vary. For instance, one may encounter a wall or a tree that prevents further navigation. Physical movement and visual perception however, are not the sole contributors to successful spatial navigation.

Place cells, discovered by O'Keefe and Dostrovsky (1971), are a specific type of neuron located in the hippocampus that aid in the perception of one's environment within spatial navigation. In a landmark study that later contributed to a Nobel Prize^3^, Moser and colleagues (Fyhn et al., 2004; Moser et al., 2017) discovered that place cells in the dorsal hippocampus had firing fields with precise spatial positioning and changes in positioning that formed a 2-dimensional “grid” representation of a subject's spatial environment. In other words, brain scans during spatial navigation (3D physical environments) revealed neural activations (2D images) that correspond precisely to the physical environment. This means that the brain may have specific neurons responsible for encoding each of the relevant objects and features (including perimeter) within one's physical environment, altogether contributing to the construction of a spatial map of one's surroundings.

A methodological constraint faced by neuroscientists studying movement is that brain imaging at the cellular level requires immobilization of subjects. How does one measure how the brain processes information during movement when the subject is unable to move during measurement? In order to offset restrictions in physical movement during brain imaging, neuroscientists rely on virtual reality (Carandini and Churchland, 2013; Stowers et al., 2017; Pinto et al., 2018).

Critically however, hippocampal place cell activation may not necessarily be consistent across navigation of real and virtual worlds, as evidenced by recent work that finds that place cell neurons fire more actively in the real world than in virtual worlds (Ravassard et al., 2013; Aghajan et al., 2015; Acharya et al., 2016).

This difference may be attributed to the reduction of navigation in VR to visual inputs and locomotion that does not account for the integration of olfactory stimuli and vestibular information that is needed in real-world navigation (Minderer et al., 2016). Given the lack of such proximal cues (olfactory, vestibular stimuli) in virtual environments, place cells may have difficulty accurately perceiving one's positionality. In VR simulations of immobilized subjects, place cells seem to keep track of a subject's relative distance along a virtual track, as opposed to encoding a position in absolute space.

While neuroscientific research about hippocampal place cells deals strictly with neural processing of spatial navigation, the revelation that place cell activations are attenuated in virtual settings relative to physical settings is an unwitting contribution to research in media psychology and human-computer interaction. Neuroscience may contribute to addressing the questions central to VR research. For instance, are VR simulations representative of our physical experience? Why do VR users experience motion sickness? In fact, the mismatch between visual perceptual information and lack of olfactory/vestibular stimuli during VR spatial navigation may be a factor contributing to VR-induced motion sickness (e.g., when one is physically sitting down while exploring a VR environment) (Langbehn et al., 2020). We will circle back to this discussion in latter sections of this work.

This all being said, these findings may be limited to the mechanisms of rodent navigation and may not generalize to humans. Due to measurement constraints most hippocampal place cell research is conducted on rodents and primates (Rolls and Wirth, 2018). Distinguishing between primates and rodents, however, Rolls and Wirth (2018) found that primate hippocampal spatial neurons activate to where a primate is looking (allocentric visual perception) (Rolls and O'Mara, 1995; Georges-François et al., 1999) in both virtual and physical environments (Wirth et al., 2017), whereas rodent hippocampal neurons activate corresponding to their positionality (ideothetic visual perception) (McNaughton et al., 1991; Jeffery et al., 1997) in a process known as “dead reckoning”^4^ (McNaughton et al., 1991). In other words, rodents navigate their environment based on a calibration of their current position relative to previous positionality. Thus, rodents seem to rely less on visual perception and more on a mental map of a spatial environment, lending literal support to the idiom, “even a blind squirrel finds a nut in awhile.” Taken together, the literature on hippocampal place cells seem promising yet with many questions unanswered. Prominently, in line with Ravassard et al. (2013), do human place cells differ in activations in VR than in the real world? This question is contingent on the integration of (a) precise hippocampal measurement, (b) free-moving navigation, and (c) human subjects in VR. While more mobile forms of brain-imaging (e.g., EEG and MEG) have been used to study hippocampal activity (Pizzo et al., 2019), it is currently not feasible to investigate hippocampal activity at the cellular level (e.g., place/grid cells).

While not specifically measuring neural activity, the following further unpacks the social perception of movements, namely the correspondence between socially meaningful human and avatar movements.

### 3.2. Social Perception of Movement

Research on the social perception of human movement cues can be traced to the Heider-Simmel simulation (Heider and Simmel, 1944), where individuals tend to attribute human-like qualities (i.e., anthropomorphize, theory of mind) to moving inanimate objects (e.g., geometric shapes).

Modern avatars in virtual environments and VR may then be understood as extensions of the Heider-Simmel geometric shapes. Like the geometric shapes of the original Heider-Simmel simulation, avatars are themselves geometric shapes, namely collections of polygons, that are not necessarily any more “human” than simple geometric shapes. Of course, the visual closeness of modern avatars to human likeness, or greater anthropomorphism, may contribute to a greater sense of “a psychological state in which the virtuality of experience is unnoticed” (Lee, 2004, p. 32). Anthropomorphism notwithstanding, the *virtuality* (Lee, 2004) of avatars and geometric shapes are one and the same.

The impact of the Heider-Simmel simulation on virtual avatar simulations today is the understanding that the attribution of human-like qualities to geometric shapes is largely credited to the *movement* generated by these shapes. To that end, social perception of movements aims to delineate how humans draw social meaning from particular types of movements.

To address the social perception of movements, we first focus on the motivation systems underlying movement, before shifting to a mechanistic typology of understanding human movement.

### 3.3. Approach-Avoidance Motivations

Approach and avoidance have long been understood as the fundamental building blocks of human behavior (Miller and Dollard, 1941; Miller, 1944). For instance, the approach-avoidance conflict is one of the most fundamental concepts in social psychology (Lewin, 1935; Miller, 1959) and is colloquially understood as the "pros and cons" of every decision. This work was followed with an idea that approach and avoidance were managed by distinct nervous system structures and distinct neural substrates (Miller, 1944; Schneirla, 1959), with arguably the most prominent theory being the interaction between a behavioral approach system (BAS) and a behavioral inhibition system (BIS) introduced by Gray and Smith (1969). The BAS governs sensitivity to rewarding cues and stimuli, whereas the BIS governs sensitivity (i.e., avoidance) to punishment and threat cues (Gray, 1981).

Approach-avoidance systems may even be observed among single-celled organisms (Schneirla, 1959), suggesting that approach and avoidance responses may be observed across all biological species. In microscopic videos, single celled organisms (e.g., protozoa) are observed to physically approach weak light (e.g., reward) and physically avoid strong light (e.g., threat). Unlike the physical manifestations approach-avoidance observed in these rudimentary organisms, human approach-avoidance is traditionally measured as a psychological construct (Carver and White, 1994) rather than as physical movement.

In line with such physical manifestations of approach and avoidance among rudimentary organisms, can human movement represent psychological approach and avoidance systems? For instance, could approach systems (e.g., BAS) be responsible for forward movement as opposed to a reversing movement?

### 3.4. Social Kinematics

The lack of accounting for physical movement in traditional psychological research on human approach-avoidance systems may have been attributed to measurement constraints. In VR, however, such physical movements are a natural and fundamental feature, warranting consideration in approach-avoidance research. In games and VR, avatar representations can be understood as visual outputs of computational data. In the context of movement and physics of avatars, every position and movement of an avatar represents a visual output based on kinematic data (e.g., position, trajectory, speed). In classic mechanics, kinematics refers to mass and acceleration explaining the geometric nature of movement, whereas dynamics refers to force explaining the cause of the movement. Expanding kinematics into a social context, we introduce the concept of *social kinematics*, which we define as socially meaningful outcomes associated with the geometric nature of human movement according to distance, trajectory, and speed (Jeong et al., 2018). More specifically, social kinematics refers to psychological inferences humans draw from precise measurable units of the combined planes and axes of movements of users' (virtual) bodies (e.g., legs, arms, heads, hands), the changes in these units of measurements (representing directional movement), and the rate of those changes (representing speed of movement). In VR, users experience a virtual world 3-dimensionally, with 6 measurable degrees of freedom (DOF) in spatial representation. In addition to the 3 DOF (*x, y*, and *z* axes; pitch yaw, and roll) of rotational movement specific to head movements, modern VR adds 3 additional DOF of translational movement along the *x, y*, and *z* axes. As VR systems are equipped with sensors and accelerometers on headsets as well as controllers, VR systems combine kinematic fidelity of hand (e.g., waving) and head (e.g., nodding) with a sense of shared physical (virtual) space.

While legs, arms, head, and hands have unique mechanics that impact their respective kinematic ranges, the current work focuses on the most basic of movements relying solely on the *x* and *y* axes, namely locomotion, or user navigation of space. In simplest terms, locomotion may be understood as 2-dimensional movement across a 2-dimensional plane, as seen in the Heider-Simmel simulation. Much like the microscopic 2D representations of single celled organisms representing approach-avoidance, the literal approach (forward) and avoid (backward) movement in human locomotion may be examined in terms of approach-avoidance systems^5^. Indeed, recent work has applied approach-avoidance systems to user navigation of virtual spaces in VR with outcomes, such as interest, attention, and curiosity, which may in turn impact social presence (Lee et al., 2019). This landmark work introduces player movement as a measurable phenomenon, and VR and virtual environments as an innovative methodology for social science outcomes. Generally, as VR takes place within finite virtual environments, VR experiences represent a negotiation of spatial politics (Pierce et al., 2011; Jones et al., 2014), departing from other modalities where space is not a finite resource. As such, the use of locomotion and co-navigation of a virtual environment among users vis-à-vis one another represents a uniquely critical aspect of social presence.

## 4. Proteus Effect, expanded

Having established our focus on kinematics within VR, we now circle back to the central purpose of this work: an expansion of the Proteus Effect. To reiterate, the Proteus Effect is an effect that is reliant on avatar appearance as movement-based representations of identity. That said, relatively less work has focused on the action affordances of avatars as additional idealized representations of identity. For instance, participants embodying more “muscular” avatars exhibit higher grip strength (Kocur et al., 2020b).

At the core of the current proposal to expand the Proteus Effect are two questions: First, in line with Kocur et al. (2020b) how does the appearance of an avatar correspond with the action affordances of the avatar? Here, we refer primarily to movement affordances, but we do recognize that virtual affordances are not limited to purely human movements. For instance, a mixture of game-based fantasy (e.g., flying, casting spells) may blend with real-world human affordances (e.g., walking, running). Second, to what degree do the physical actions of the user correspond with the virtual actions of the avatar?

We propose a systematic exploration into the role of movement affordances in VR and virtual environments, both in terms of (a) social perceptions (by others) and (b) idealized perceptions (of oneself). Movements in VR may range from high fidelity direct representations (e.g., motion tracking) to an interaction with game-based fantasy. For instance, boxing games on VR represent the former, as physical and virtual movements (e.g., a punch) have a direct correspondence. Not only is this movement high in fidelity (an actual punch is a virtual punch), but the physical punch is an actual human affordance. On the other hand, social VR platforms (e.g., VRChat) have a teleport function that is not necessarily a high fidelity translation of actual movement to virtual movement. In other words, instead of a punch being represented as a punch, teleporting in VR is achieved with a controller button press. Along this vein, movements may be categorized according to representativeness to one's actual kinematics relative to one's affordances.

**Figure 1:**
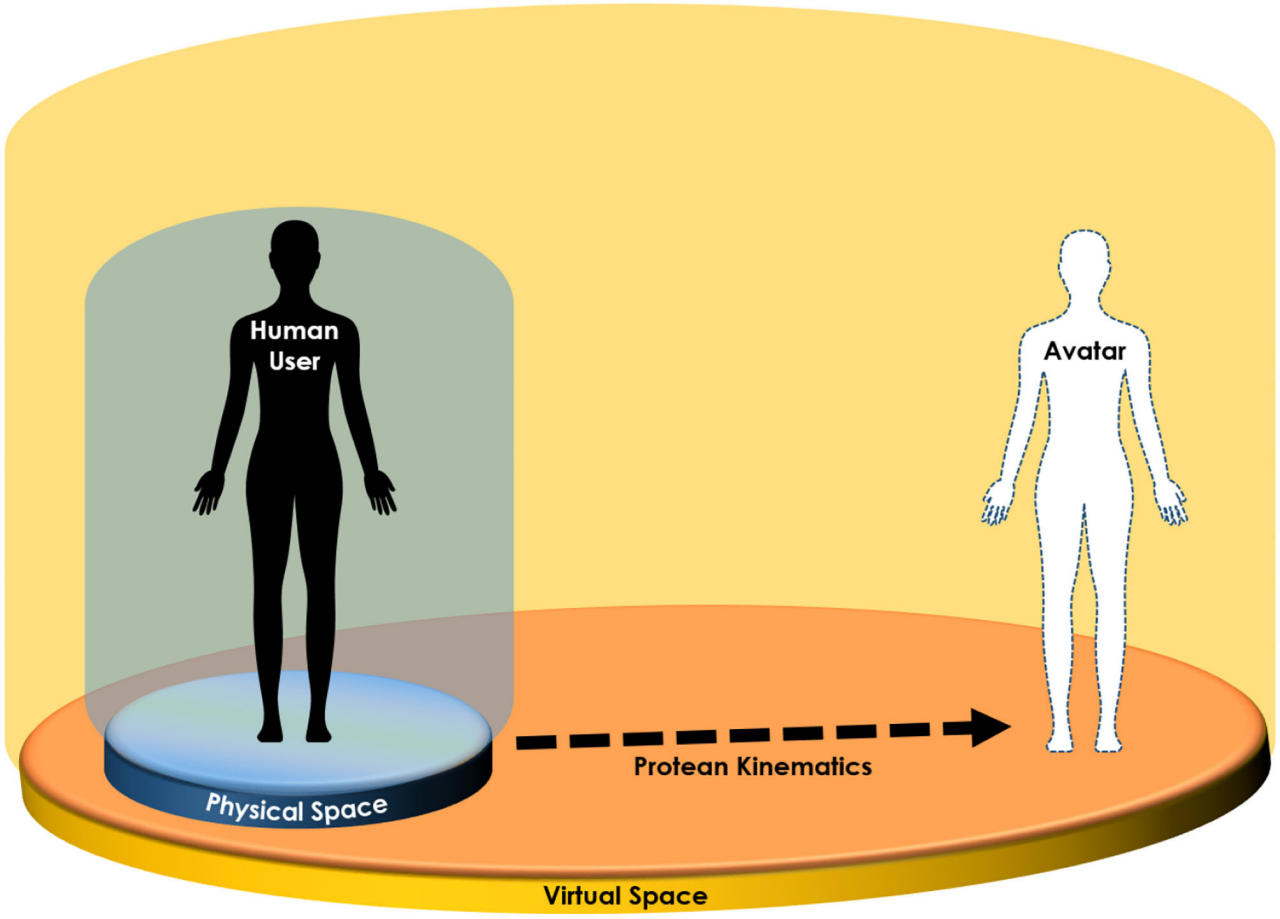
Protean kinematic refers to the idealized virtual movements that can take human users beyond the constraints of their physical affordances (e.g., space).

In line with the above, we propose expanding the Proteus Effect to account for movement affordances vis-à-vis user and avatars, which we refer to as *Protean kinematics* (Figure 1).

### 4.1. Typology of Protean Kinematics

**Figure 2:**
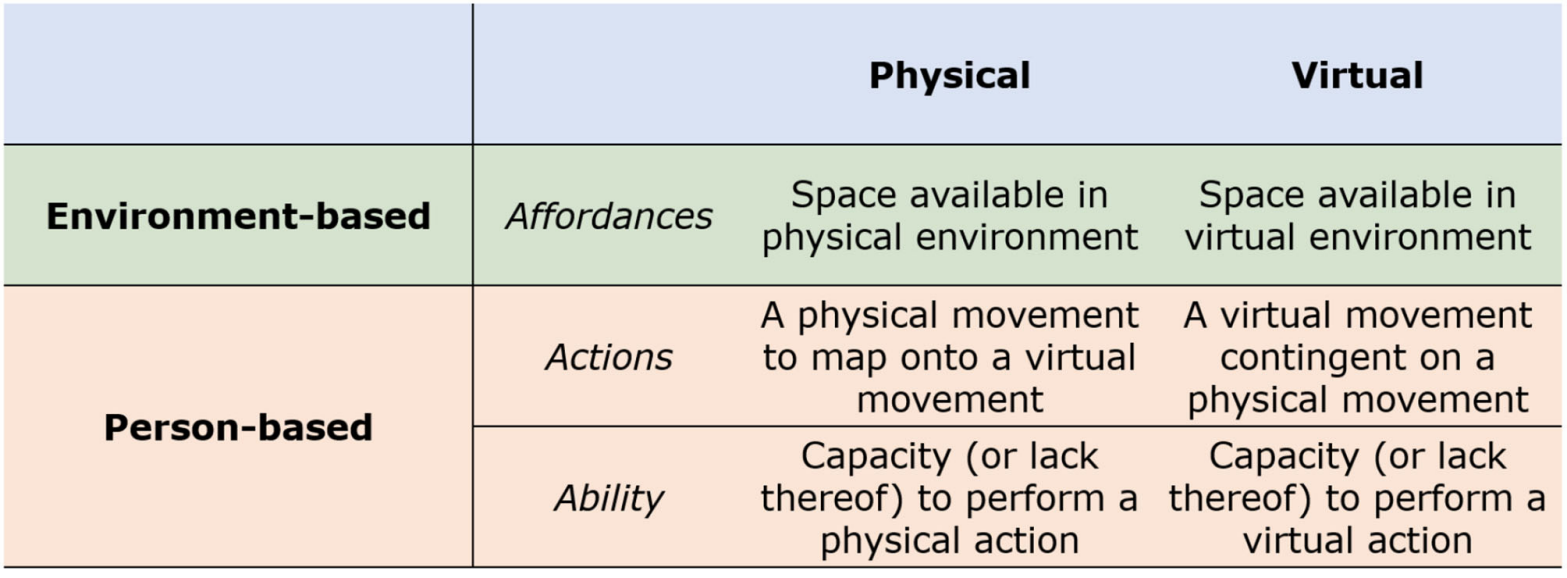
Typology of Protean kinematics, which refers to the intersection of physical affordances/actions and virtual affordances/actions. Here, we depict affordances as space, physical actions as human actions, and virtual actions as avatar actions.

Drawing from Lewin's argument that human behavior is a function of both “persons” and “environments” (Lewin, 1936, 1943), our typology of Protean kinematics (see Figure 2) begins with a distinction between what is possible given one's environment (*affordances*), and what is acted upon by the person (*actions*). Along this vein, we may interpret human behavior in virtual reality as a continuum of movements along the two axes of affordances and actions in the real-world (physical) and in the virtual world. In other words, affordances and actions may be distinguished between human movement in physical environments and avatar movement in virtual environments.

In the current typology, affordances refer to the movement constraints of one's ability (person-based) and one's surroundings (environment-based). *Person-based affordances* refer to both the physical capacity of the particular user (e.g., the capacity to walk among able-bodied individuals), or the virtual capacity of an avatar (e.g., such as the capacity to leap 40 feet in the air). Person-based affordances are central to traditional applications of the Proteus Effect, such the degree to which the appearance of an avatar may or may not correspond with the actions of the human user Kocur et al. (2020b), or studies that examine plasticity of virtual and physical body ownership (Piryankova et al., 2014). *Environment-based affordances*, on the other hand, refer to the constraints in one's physical environment (e.g., a limited play space), or the constraints in one's virtual environment (e.g., the perimeter of a virtual map). Altogether, different degrees and levels of person-based and environment-based affordances in VR modify and bend common assumptions of 1-to-1 correspondence between physical and virtual actions, as seen in natural mapping technologies (Birk and Mandryk, 2013; Vanden Abeele et al., 2013), such as Microsoft Kinect.

### 4.2. Person-Based Actions

VR presents a unique psycho-physiological quandary of physical inputs and virtual outputs. On one hand, the degree of overlap and correspondence between physical inputs and virtual outputs may be understood as a measure of *validity* (Godoy et al., 2008). That is, does the device (e.g., headset, hand controller) reliably measure human kinematics as assumed within natural mapping technologies (Birk and Mandryk, 2013; Vanden Abeele et al., 2013)? On the other hand, it is worth understanding the conditions for demanding high reliability, namely considering a potential mismatch between goal-driven intent (e.g., a greeting “hello” wave) and perception (e.g., a dismissive wave). We first proceed with an *assumption* of correspondence between physical behavior (inputs) and virtual behavior (outputs), which we refer to in our typology of Protean kinematics as *person-based actions*. That said, certainly a 1-to-1 correspondence would not be particularly protean! Our subsequent section on affordances will address this issue, but we begin by addressing correspondence.

The transfer of physical human movement to virtual avatars is certainly not a novel concept. Broadly speaking, human movement behavior has long been of interest to scholars examining natural mapping (Birk and Mandryk, 2013; Vanden Abeele et al., 2013), intelligent virtual agents (Gratch et al., 2002; Thiebaux et al., 2008; Marsella et al., 2013; Kucherenko et al., 2021), VR-based gesture tracking (Won et al., 2012; Christou and Michael, 2014), and pose estimation of anatomical keypoints (Andriluka et al., 2010; Pishchulin et al., 2012; Cao et al., 2017). Natural mapping motion capture systems, which generate virtual avatar representations based on physical human behavior, vary from 3D pose estimation [See (Wang et al., 2021) for a review] to facial expression sensors (Lugrin et al., 2016). A commercial example of such systems is the Microsoft Kinect, which has been utilized for detecting gender (Won et al., 2012) as well as differences in avatar types (Christou and Michael, 2014). That said, given computational constraints of such systems, there are efforts to more reliably map human movement to 3D graphical representations using full-body motion tracking suits (Roetenberg et al., 2009). These motion capture systems may be integrated with wearable micro-electromechanical systems (MEMS) to promote health outcomes (Brigante et al., 2011). The cost and accessibility constraints of such systems however, have motivated the development of lower-cost alternatives using fewer sensors (Caserman et al., 2019b). A potential compromise between the accessibility (e.g., less costly) of fewer sensors and greater reliability of motion capture systems (Jeong et al., 2020) may be achieved by integrating virtual reality sensors with a combination of inverse kinematic techniques (Roth et al., 2016; Caserman et al., 2019a), pose estimation (Cao et al., 2017), and temporal convolutional networks (Lea et al., 2017). Motion tracking in VR has already been used to test homuncular flexibility (Won et al., 2015; Bailey et al., 2016; Herrera et al., 2020; Kocur et al., 2020a), as well as health-based physical activity (Hahn et al., 2020; Navarro et al., 2020).

In prior work (Jeong et al., 2020), we have proposed utilizing existing sensors native to VR headsets and controllers among users as an alternative to costlier motion tracking procedures. This involves utilizing a technique known as inverse kinematics to represent these first-person movements of users as third-person movements avatars (Roth et al., 2016). At the final step, third-person avatars “re-create” a third-person virtual representation of the first-person human movements. This step requires the use of multiple virtual cameras to attain multiple perspectives of the third-person avatar movement in order to achieve an accurate representation of depth and to avoid object occlusion. The resulting simulated avatar/character animations may then be analyzed computationally using computer vision (e.g., pose estimation), which in turn may be used to analyze human behavior in various “virtual” contexts (e.g., doctor's office, job interview, romantic date, interracial dialogue). Such virtual animations may potentially approach hybrid avatar-agent systems of human-human interactions mediated by avatars and artificial social intelligence (e.g., to moderate intercultural conversations) (Roth et al., 2015).

We pause to reiterate the emphasis of the current work on the kinematic representations in VR. Within Protean kinematics, person-based actions assume or aim for a correspondence between physical and virtual actions. That said, the degree of correspondence between physical and virtual actions is constrained by both person-based and environment-based affordances. Virtual person-based affordances (avatar movements) are so-defined by the game developers, while physical person-based affordances (human movements) represent the physical capacity of an individual. For instance, differences in body size of an avatar will place a natural constrain on range of movements, which may be associated with different perceptions of meaning and intent. While we intend to expand our investigation of such person-based affordances in forthcoming work, our subsequent references to affordances in the current work refer to environment-based affordances, or the spatial constraints of one's physical and virtual environments (see Figure 2), which we elaborate in the following.

### 4.3. Environment-Based Affordances

Replicability and methodological reliability are certainly paramount to the development of science. What is the role of virtual reality and virtual environments in such scientific development? Virtual environments may afford an emergence of a uniquely virtual form of validity that measures the correlation between virtual and real-world behavior (Godoy et al., 2008). Ultimately, virtual environments may warrant a shift in the use of traditional experimental designs in the social sciences into more systematic designs that are representative of the real-world social situations (e.g., interpersonal conflict) and outcomes (e.g., promote vaccination) they reference (Miller et al., 2019a,b). That said, virtual environments are not a one-size-fits-all panacea for all science. Indeed, recent a meta-analysis rejected a longstanding misconception of VR as a “device of empathy” (Martingano et al., 2021). Another misconception of VR research is that all types of VR affords a complete perceptual embodiment, such as a window to another world and another person's perspective and experience. The former may be possible in a “television” model of VR in 3DOF experience (e.g., 360 VR), while the latter would require the full 6DOF range of human head rotation.

While virtual human behavior may correlate to real-world behavior (and vice-versa), a central aim in VR research should be to establish the kinematic fidelity, or reliability between physical and virtual behavior. The correspondence between physical and virtual behavior warrants the consideration of environment-based affordances in addition to the person-based affordances taken into account in natural mapping and motion tracking. On the contrary, a blanket argument that physical behavior corresponds to virtual behavior would assume that the physical inputs may be occurring in a vacuum or in infinite space.

**Figure 3:**
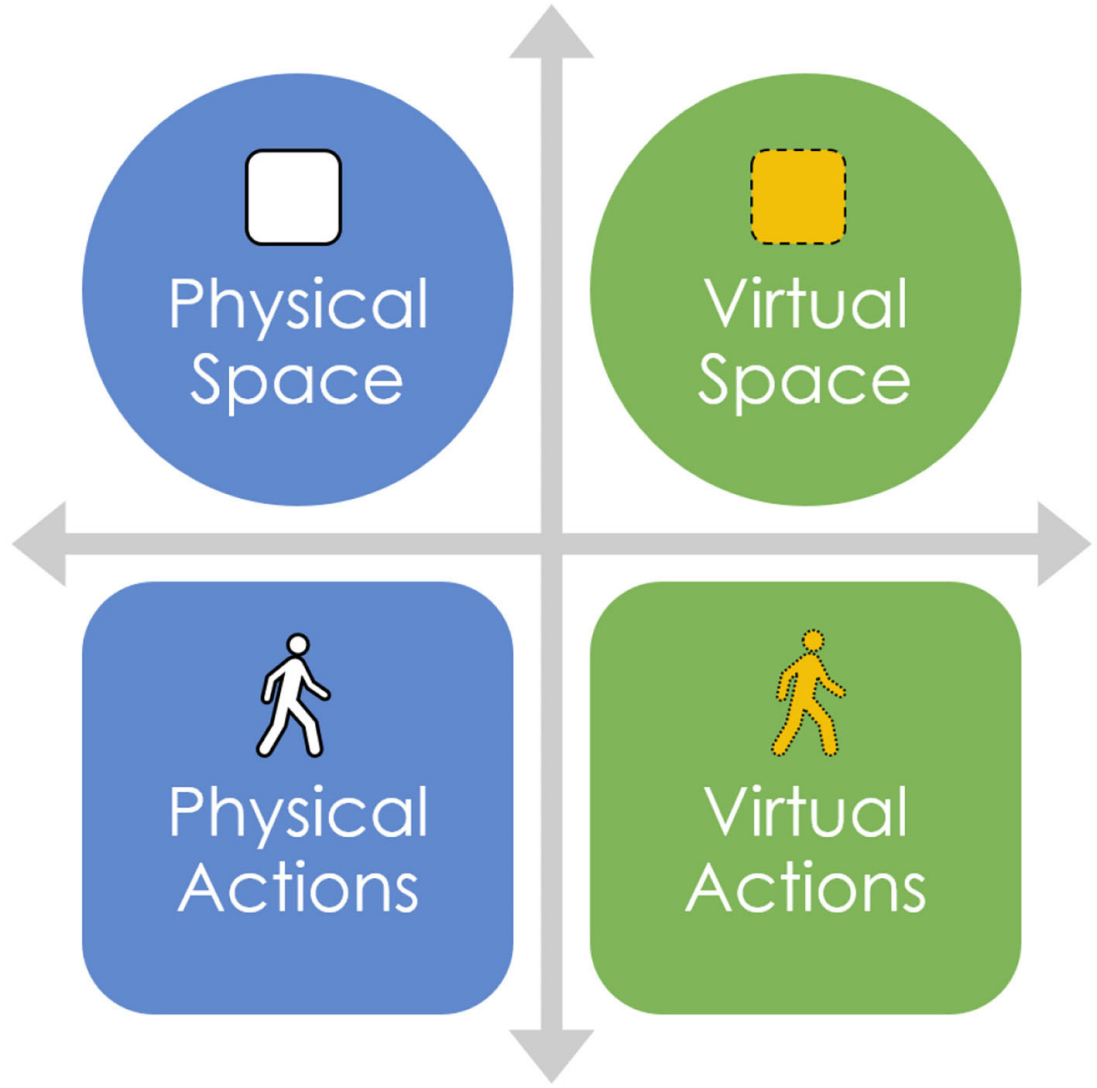
Protean kinematics occurs at the intersection of physical affordances/actions and virtual affordances/actions. Here, we depict affordances as space.

Indeed, a requisite consideration to the real-world correlates of virtual avatar/agent outputs in VR is—quite literally—*space* (Figure 3). VR movement is constrained by the availability of free physical space the user has to navigate, regardless of infinite virtual space. While physical space is typically not a major concern for research lab environments dedicated to providing obstacle-free “play areas^6^,” casual users rarely have the opportunity to navigate (e.g., walk around) a truly physical environment that is “true to scale” with the given size of a virtual environment. When constrained by space, VR users rely on a *stationary* play area that assumes a seated and immobile position that relies more heavily on a teleport function for locomotive navigation throughout a virtual space^7^. As such, VR does not necessarily assume high fidelity of kinematics from physical inputs (by humans) to virtual outputs (by avatars). Indeed, users consistently underestimate depth perception in VR (Renner et al., 2013; Maruhn et al., 2019). This lack of correspondence between the physical inputs and virtual outputs warrants investigating the intersection of affordances-actions across physical and virtual environments.

Here, we circle back to our earlier discussion of place cells/neurons in the brain's hippocampus. VR devices are designed as (head-mounted) displays (i.e., screens, monitors) that provide visual representations of virtual spaces (environment) larger than what is physically available. This visual information from the display, however, may not necessarily correspond with the combination of olfactory and vestibular sensory information (or lack thereof) required in traditional spatial navigation. In fact, the absence of these additional sensory inputs may be responsible for the notorious cybersickness users experience in VR [See Weech et al. (2019) for review of the relationship between cybersickness and presence]. Although a systematic analysis of cybersickness is beyond the scope of the current work, the conditions that are associated with VR cybersickness are relevant to the intersection of physical and virtual spatial navigation. For instance, cybersickness has been examined among subjects that are sitting (Palmisano et al., 2017) and standing still (Palmisano et al., 2018). Sitting and standing are both physical orientations that are stationary (e.g., no locomotion involved), and would warrant the utilization of virtual locomotive techniques to navigate in a virtual space, namely *steering* and *teleporting*. Clifton and Palmisano (2020) recently examined potential interactions between physical body positioning (sit/stand) and virtual locomotive techniques (steer/teleport), reporting greater cybersickness for stand-steer conditions than others, least cybersickness for sit-steer conditions than the teleport conditions. The authors also report that cybersickness progressively increase over time in teleport conditions, but level off after an initial spike during sickness (Clifton and Palmisano, 2020). This may be attributed to the smoother contiguity of previous scenes and subsequent scenes during steering-based locomotion relative to teleport-based locomotion. Among the above conditions, the standing conditions is of most relevance to the current work. In a growing body of work, Palmisano and colleagues have attributed cybersickness to lack of postural stability, measured by fluctuations in center-of-foot pressure (Palmisano et al., 2014, 2018). Indeed, merely standing-still requires engaging multiple physiological and neurological systems to achieve and maintain balance.

Recently, VR has been utilized to rehabilitate the gait and balance challenges experienced by Parkinson's patients (Canning et al., 2020). That said, is the design of VR devices able to accurately simulate the requisite conditions for physical walking, or locomotion? The somewhat disparate domains of rehabilitation, cybersickness, and redirected walking are all united by the lack of correspondence between physical and virtual spatial navigation. Taken together, VR locomotion must account for the incongruity between simulated locomotion in the absence of (corresponding) physical locomotion and the manner in which the brain processes physical locomotion.

The above however, is assuming a model of physical inputs and virtual outputs fully dependent on corresponding physical inputs. An alternative approach is to consider goal-directed (e.g., approach-avoidance systems) virtual outputs with the use of physical inputs as tools for achieving a virtual goal. In line with the Proteus Effect, different avatar identities may elicit different types of movements both physically in-person, as well as virtually in-game. For instance, Kocur et al. (2020b) demonstrate that identification with a “strong appearing" virtual avatar may elicit greater physical strength (e.g., grip strength). Of course, this physical-to-virtual correspondence may be constrained at the game development level. For instance, if the maximum virtual output is generated by gripping a controller with 10 pounds of force, then gripping a controller with 100 pounds of force will have no net difference in the virtual output. Alternatively, in keeping with our focus on locomotion, greater identification with an aggressive avatar may elicit a more direct (as opposed to circuitous) locomotive trajectory and velocity toward a virtual target/goal (e.g., another avatar).

Regardless of the nature of movement, VR movement tends to operate in this goal-directed manner. For instance, the teleport movement is the clearest example of goal-directed behavior in VR that relies minimally on corresponding physical inputs. This represents a developmental paradox of VR: on one hand, the lack of available physical space as well as the onset of cybersickness compels users to use virtual locomotive techniques that require minimal physical movement such as teleporting and steering; on the other hand, kinematic fidelity between physical and virtual locomotion may be required. Ultimately, hippocampal place cells, vestibular inputs, olfactory inputs, visual-spatial inputs, as well as postural stability may all require physical locomotion (actual walking) to afford the virtual representation of VR locomotion.

Having established space as central to environment-based affordances, we continue our proposal to expand the Proteus Effect by discussing the challenges of translating locomotive kinematics from physical to virtual environments.

## 5. Measuring Locomotive Kinematics

In the following, we continue to discuss the challenges of translating physical kinematics to virtual kinematics in VR. Kinematic fidelity would assume a seamless correspondence between physical movements as inputs and virtual movements as equivalent outputs (e.g., a physical “thumbs-up” and a virtual “thumbs-up”). Kinematic fidelity in VR is particularly operative in the case of head rotations and arm movements, but less so for locomotion, where users may utilize a number of virtual locomotion techniques (e.g., teleporting, steering) in lieu of the physical locomotion (which take up considerable physical space/affordances). Two main constraints to kinematic fidelity in VR locomotion may be (a) the absence of leg sensors, and (b) calibrating physical units of measurement (e.g., meters) to virtual units of measurement (e.g., pixels). For instance, computer mouse cursors are calibrated with a specific sensitivity for each computer user. Certainly, one inch of physical mouse movements does not necessarily output one inch of mouse cursor movements. In an attempt to explain the mechanistic process of transforming physical movements into virtual movements, we focus specifically on the locomotive navigation of users and avatars in a virtual space, and exclude other forms of movement, such as head rotations and arm movements. Although head rotations and arm movements rely on VR sensors that are unavailable to measure leg movements on most commercial VR systems, it is worth noting that meter-pixel calibration issues remain for all types of VR movement. As we elaborate below, the calibration of physical-to-virtual units of measurement warrants a perspective grounded in image processing (i.e., computer vision), where sequences of movements are measured as sequences of images, otherwise known as frames.

### 5.1. 3D to 2D Transformation

In examining the transformation (i.e., natural mapping) of physical movements onto virtual movements, we first consider the dimensionality of physical space. Naturally, our experience of the physical world is 3-dimensional and game engines (e.g., Unity) may provide corresponding options to view active layers of 3D planes to provide a visual reference point relative to the objects on a layer (i.e., scene). This *active layer plane* (i.e., ground plane) may be understood as the floor in the physical world. Working in 3D games and modeling however, requires the cursor to snap to a 3D location. When no other 3D snap is active (the cursor is not snapping to 3D geometry), the cursor still needs to snap to a 3D location in order to provide a reference point to the 3D object (e.g., an avatar). This 3-dimensional plane is known as the *working plane*. The working plane is what allows an avatar to move across 3-dimensional space in VR environments.

However, 3-dimensionality is not necessarily a requirement when focusing strictly on locomotion. Locomotion may be understood purely as a human or agent navigating throughout a given 2-dimensional space. Here, we are disregarding characteristics of movement that invoke 3-dimensionality, such as gait style. As referenced earlier, this 2-dimensional conceptualization of locomotion aligns with the original Heider-Simmel simulation, where geometric shapes move throughout 2-dimensional space. As such, projecting a 3D space into a 2D space affords a strict focus on locomotive kinematics within the virtual space.

**Figure 4:**
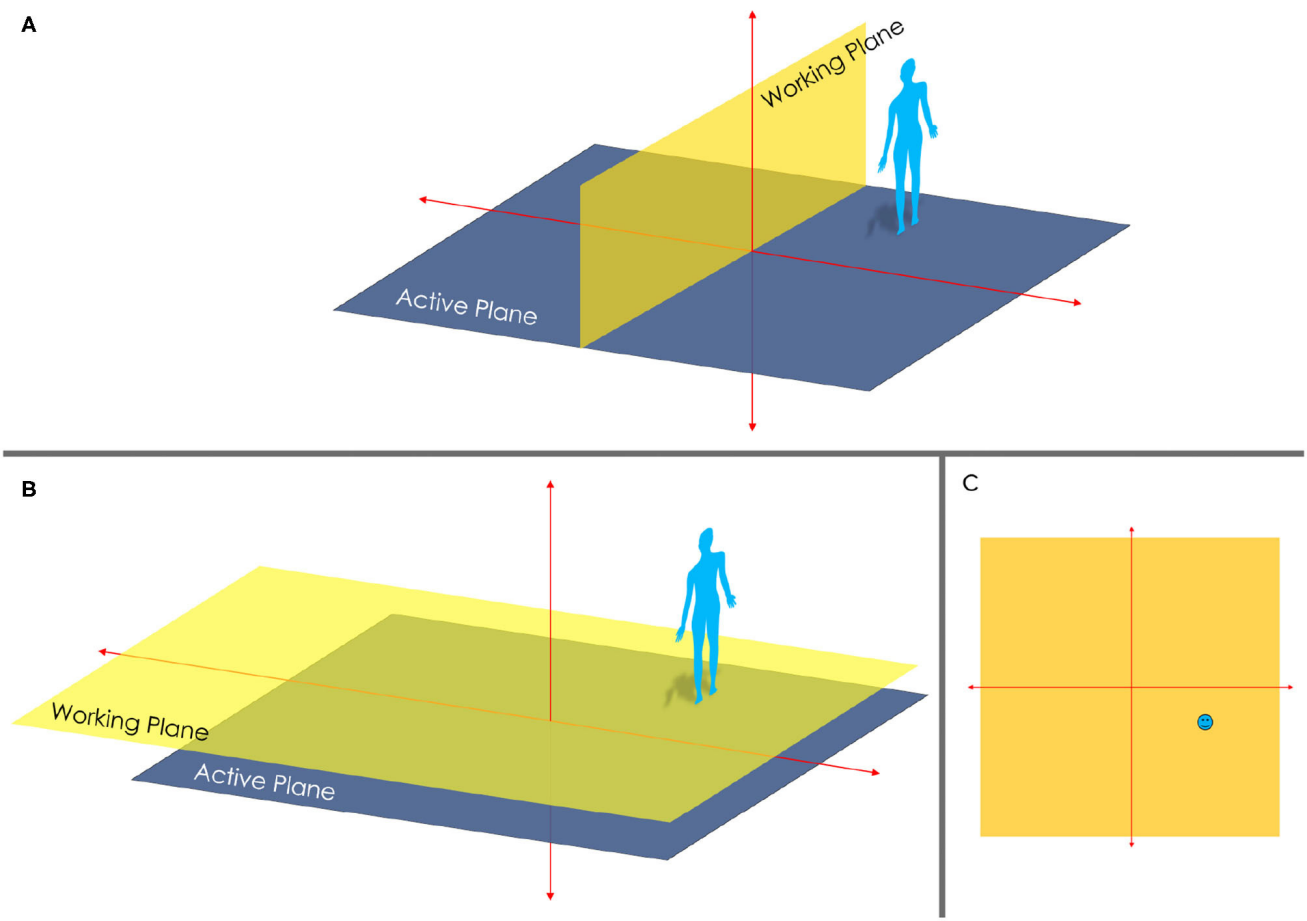
**(A)** depicts the distinction from a working plane and an active plane in 3D. **(B)** depicts the transposing of the working plane so that it is parallel to the active plane. This is what happens when transforming a 3D image into 2D. **(C)** depicts the outcome of transforming a 3D image into 2D.

The first step to transforming physical world movements to virtual movements requires projecting 3-dimensional (3D) space into a 2-dimensional (2D) “image space.” Critically, this projection of 3D space into a 2D space involves rotating the active working plane to become parallel to the working plane (e.g., ground), thus degrading the projection into a planar projection (Figure 4).

### 5.2. Physical to Virtual Units of Measurement

Working in 2D, the next step is to convert 2D representations of physical movements onto 2D representations of virtual movements, which is achieved by observing physical movements across *x* and *y* axes in physical space, such as with a downward-facing overhead camera. Physical movement, however, is not readily converted into virtual movement because of the differences in units of measurements in physical (e.g., meters) and virtual (e.g., pixels) environments. Further, we cannot assume a consistent “conversion" of physical to virtual units of measurement because this is point of personal preference and calibration. For instance, computer mouse cursors are calibrated with a specific sensitivity for each computer user. Certainly, one inch of physical mouse movements does not necessarily output one inch of mouse cursor movements. We represent the variable scale of the conversion between the physical space and virtual space using the scalar variable, γ. The complete relationship between virtual and physical movements may be represented using the equation ${\vec{m}}_{v}=\gamma \vec{m}$, where γ is a ratio between the size of the physical space, $\vec{m}$, and the size of the virtual space, ${\vec{m}}_{v}$.

#### 5.2.1. Meter-Pixel Calibration

Beyond the personal preference of γ, the conversion of physical movements to virtual movements, the transformation of physical space to virtual spaces warrants meter (physical) to pixel (virtual) *calibration*. This calibration is represented by the formula $\frac{\underline{D}}{d}=k$ where *k* is a scalar value that can be used to model the projection on the 2D space. *k* must be calibrated using the physical distance between two physical landmarks (e.g., tape on ground), denoted *D* in meters, which are in turn used to determine the image-based distance between the two landmarks, denoted *d* in pixels. The complete transformation of the physical space to the virtual space is represented as follows: $\vec{m}=k\vec{a}$ where $\vec{m}$ represents the physical space and $k\vec{a}$ represents the calibrated (*k*) image of the image space ($\vec{a}$) (taken from a downward facing overhead perspective).

#### 5.2.2. Virtual Locomotion

**Figure 5:**
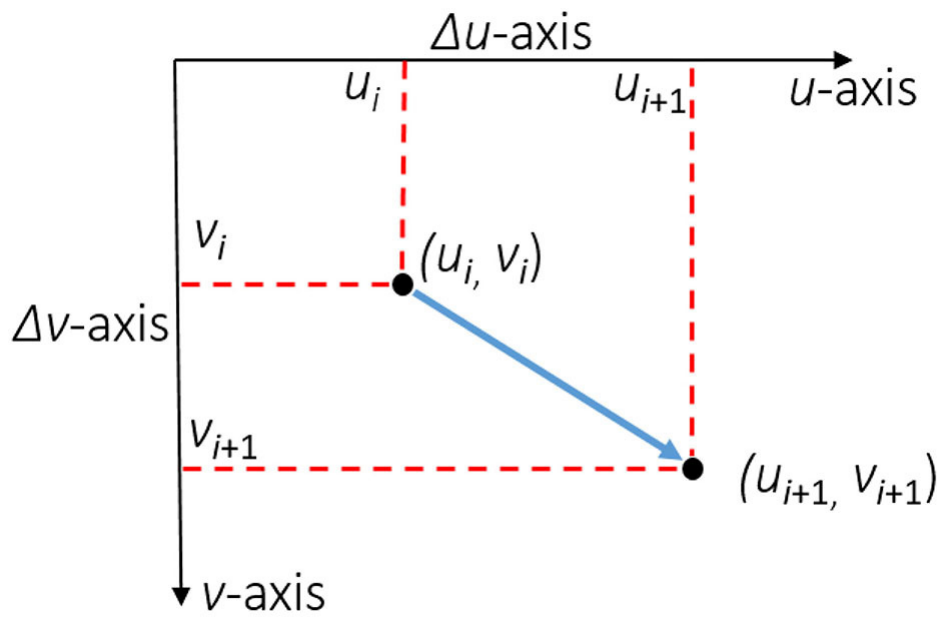
Here we depict where movements in 2D image space. *u* and *v* represent coordinates in the image space at a specific prior time point (e.g., before movement), and *i* is the frame index in the image flow. *u* and *v* are image-based coordinate representations of vector spaces, represented by Δ*u* − *axis* and Δ*v* − *axis*. Each successive point is represented by *P*_*i*+1_ = (*u*_*i*+1_, *v*_*i*+1_).

Now that the physical space is transformed into an image space (2D), we can represent locomotion of individuals (or avatar/agent representations of individuals) within this space (Figure 5). As alluded earlier, the process of converting physical to virtual kinematics borrows from traditions in image processing, where motion is represented as sequential coordinates from point to point (e.g., frame by frame): *P*_*i*_ = (*u*_*i*_, *v*_*i*_) where *u* and *v* represent coordinates in the image space at a specific prior time point (e.g., before movement), and that *i* is the frame index in the image flow. While *x*-axis and *y*-axis are physical world coordinates, *u* and *v* are image-based coordinate representations of vector spaces, represented by Δ*u*−*axis* and Δ*v*−*axis*. Each successive point is represented by *P*_*i*+1_ = (*u*_*i*+1_, *v*_*i*+1_). *P* assumes frame by frame processing, which should suffice for a wide range of human movements^8^.

#### 5.2.3. Virtual Kinematics

To reiterate, kinematics is a branch of physics derived from classical mechanics that describes the geometry of motion. Having explained the preparation of movement data as image-based coordinate representations of sequential frames and subsequent frames, the following addresses how to compute some of the notable kinematic quantities, namely trajectory, velocity, and distance.

##### 5.2.3.1. Trajectory

Assuming that there are only 2 time points (or frames) of movement, we would represent the *trajectory* of movement between these 2 points as $\vec{a}=\left(u_{2}-u_{1},v_{2}-v_{1}\right)$, where $\vec{a}$ represents the trajectory of movements in coordinates across 2 points along the first time point, (*u*_1_, *v*_1_) and the second time point, (*u*_2_, *v*_2_) (Figure 5).

##### 5.2.3.2. Velocity

Velocity of movement would simply be calculated by dividing the trajectory of movements by Time, $\text{Vel}=\vec{a}/\text{T}$.

##### 5.2.3.3. Distance

Finally, in order to compute the distance between the two points, we use the ℓ^2^-norm, otherwise known as the Euclidean norm, which is used to compute vector lengths in Euclidean space using the square root of the sum of the squares of the values in each dimension (Weisstein, 2000; Li and Jain, 2015):

(1) $$\begin{array}{r} ||a||=\sqrt{\left(u_{2}-u_{1}\right)\times \left(u_{2}-u_{1}\right)+\left(v_{2}-v_{1}\right)\times \left(v_{2}-v_{1}\right)} \end{array}$$

## 6. Discussion

At its core, the current work focused on a conceptualization of human-agent interaction, namely the relationship between human interlocutors and the agents that represent them. Broadly defined, avatars may be understood as virtual (i.e., visual) representations of agent simulations of human interlocutors. In the narrower context of virtual reality, virtual representations rely on physical kinematic inputs with varying degrees of fidelity. For instance, VR head rotations represent movements that involve a high degree of correspondence between physical inputs and virtual outputs. Alternatively, VR locomotion may occur fully in the absence of corresponding physical locomotion, producing a perceptual experience of walking (e.g., teleporting, steering) without the physical act of walking. The current work investigated the complex relationship between physical and virtual movement in two ways. First, we explicated a typology of Protean Kinematics, focusing particularly on the physical-virtual intersection of person-based actions and environment-based affordances. Second, we outlined the steps required to transform physical locomotion into virtual representations, namely by undergoing a 3D to 2D transformation, as well as by calibrating the conversion of physical to virtual units of measurement (meters to pixels).

Ultimately, Protean kinematics assumes a mismatch between the physical and virtual actions, a mismatch predicated on affordances in physical and virtual spaces, preferential sensitivity (e.g., mouse sensitivity), and calibration of physical to virtual units of measurement (e.g., meters to pixels). Such a lack of kinematic fidelity across physical and virtual experiences, as well as biological advances that underscore the significance of “actually walking” to the brain's perception of one's spatial environment introduce constraints and limitations to VR development. Insofar as VR is conceptualized as a “window” into an alternate reality (e.g., environment) for users that lack adequate physical space to navigate such virtual spaces, user experience may be considerably constrained (e.g., cybersickness). Addressing such a constraint may warrant re-conceptualizing VR development beyond purely the visual perception of image projections on screens (e.g., monitors), and incorporating additional systems, such as vestibular and olfactory inputs, as well as postural stability. Scholarly work on hippocampal place cells and grid cells indicate that beyond the visual perception of navigating an environment, our brains may require the act of walking to encode both objects within and perimeters of a given environment/space. Further, recent evidence suggests that speed of movement (e.g., running) is reflected in the firing rate of a distinct class of entorhinal neurons, knwon as “speed cells” (Kropff et al., 2015). Perhaps the simplest solution may be to utilize expansive physical environments that afford corresponding fidelity between physical and virtual locomotion. Another option may be to equip VR headsets with multi-directional thrusters (i.e., fans) that would blow physical air in the direction and strength corresponding with a virtual locomotive trajectory. Both of the above suggestions however, are quite costly in both physical space and hardware development. An alternative solution may be to abandon virtual reality altogether and to turn to mixed/augmented reality, thus affording virtual objects and actions to appear within a fully perceivable physical environment.

By introducing Protean kinematics, we present a conceptual model that blends and blurs the distinction between physical and virtual experience. The future of avatar research in VR should consider the increasingly blended nature of mediated experience. Mixed reality or augmented reality (Silva and Teixeira, 2020; Papakostas et al., 2021a,b) quite literally address the “blend" between physical and virtual experiences (e.g., objects, people/avatars, environments) (Miller et al., 2019d). In mixed reality, the hippocampus may retain normative processing of spatial navigation in one's physical environment while users flexibly engage in a variety of virtual actions. Given the potential harmony between physical environments and virtual actions, mixed reality may afford tremendous potential to improve our biological understanding of spatial navigation. That being said, mixed reality excludes the virtual environment, one of the fundamental components of multiplayer gaming and the sharing of virtual spaces (e.g., social VR).

Social perception of movement is important in considering avatars in present day and into the future given expanding use and applications of avatars beyond gaming contexts, such as by major social media platforms (Snapchat Bitmoji, Apple Memoji) and web conferencing platforms (Mozilla Hubs, Gather.Town) which are cost-efficient and have low barriers for entry. Internet users are also increasingly using avatar generating platforms such as FaceRig, and VRoid Studio, which are “face-rigging” software that generates real-time responsive avatars^9^. Meanwhile, developers continue to improve the accessibility of avatars, virtual humans, and character animations for researchers, as seen in the Microsoft Rocketbox virtual human library (Gonzalez-Franco et al., 2020) and the Adobe Mixamo character animation system^10^, both of which are readily integrated with Unity and capable of being imported into Blender.

### 6.1. Future Research

Continued innovation and rising demand for avatars warrants widespread scholarly attention from social science researchers. Avatars may be understood as virtual/visual representations of human goal-directed actions. While substantial avatar research is paid to avatar appearance (e.g., Proteus Effect), the current work focused on avatar movements, namely the complex relationship between physical and virtual kinematics. We propose future research to consider the role of kinematics in the social perception of avatar, with particular attention on the contingent physical actions driving virtual actions in VR. As noted in the current work, VR kinematics (particularly locomotion) must be understood in light of the oft-mismatched or lack of correspondence between physical and virtual environment-based affordances (e.g., space).

Within the social sciences, virtual representations of real-world situations may afford greater generalizability (Miller et al., 2019a,b), which may contribute to scientific replicability. By combining virtual flexibility and realism with culturally-nuanced representations of real-world situations and circumstances (Kim et al., 2021), VR affords investigating the dynamics of human behavior for real-world outcomes (e.g., decision-making). Further, the approach introduced in the current work may be supplemented using computer vision and machine learning techniques that afford analysis of real-time sequential behavior, such as gestures (Jeong et al., 2020). Although the current work focused on 2-dimensional locomotion, we will address the application of Protean kinematics on 3-dimensional arm gestures and head movements in forthcoming work.

The mismatch between physical inputs and virtual outputs experienced in Protean kinematics may not necessarily negatively impact VR user experience. Rather than kinematic fidelity between physical and virtual movements, VR movements may be better understood as goal-directed behavior (e.g., approach-avoidance motivation systems). VR behavior can be understood as a mouse cursor that does not necessarily correspond precisely with a physical computer mouse or trackpad. As demonstrated by Won et al. (2015), imperfections in correspondence between physical inputs and virtual outputs demonstrate the homuncular flexibility of the human mind. Ultimately, Protean kinematics may be conceptualized as a form of *presence*, a psychological state in which the virtuality of movement is unnoticed (Lee, 2004).

All this being said, we would be remiss to not discuss the ethical implications of motion tracking in VR (Miller et al., 2020; Carter and Egliston, 2021). Indeed, the platformization of VR (e.g., Oculus) is of grave concern to many VR users and researchers (Egliston and Carter, 2020). At this point, we reiterate the critical significance of open science among researchers of technology and platforms. With the possibility for ethical abuse and scientific malpractice, researchers' steadfast commitment to maintaining scientific transparency and openness is critical (Open Science Collaboration, 2015; Bowman and Keene, 2018; Lewis, 2020).

### 6.2. Conclusion

In the current work, we discuss the potential fallacy in emphasizing kinematic fidelity in VR as the perceptual experience of VR environments and users does not (yet) correspond with the perceptual experience of the physical world and people. We have also presented connections of VR kinematics to neurological understanding of spatial perception and motion perception, as well as outlining.

Our explication of Protean kinematics introduced its grounding in physics (i.e., kinematics), a typology of physical/virtual actions and affordances, and a computational model describing the physics and geometry underlying the intersection of physical and virtual experience. This process requires understanding calibration based on physical to virtual units of measurement (meters to pixels) as well as calibration based on personal preference (e.g., mouse sensitivity).

This work contributes to avatar embodiment with both a theoretical (typology) and methodological (physics-based measurement) approach to understanding the complex blend of physical inputs and virtual outputs that occur in VR (Won et al., 2015). Offered is a novel method of utilizing the unique blend of physical and virtual kinematics in VR for potentially reliable measures of goals and motivation (Lee et al., 2019).

Taken together, the current work introduces a framework for re-conceptualizing behavioral measurement. Physical behavior is understood as distinct from virtual representations of such behavior. While there has been evidence of behavioral consistencies (i.e., validity) across virtual and real contexts, the current work proposes a blended approach to virtuality by exploring the intersection of physical and virtual movement in VR. Moving forward, media researchers may consider moving beyond conceptualizing physical experience as non-virtual and virtual experience as non-physical.

## Author Contributions

DJ, SK, and LM developed theory and literature review. JX developed literature review. DJ developed locomotive kinematic measurements and produced figures. All authors contributed to the article and approved the submitted version.

## Conflict of Interest

The authors declare that the research was conducted in the absence of any commercial or financial relationships that could be construed as a potential conflict of interest.

## Publisher's Note

All claims expressed in this article are solely those of the authors and do not necessarily represent those of their affiliated organizations, or those of the publisher, the editors and the reviewers. Any product that may be evaluated in this article, or claim that may be made by its manufacturer, is not guaranteed or endorsed by the publisher.

## Footnotes

^1^For example, dunking in a basketball video game, traversing a wall in Fortnight, and teleporting in VRChat each represent unique movement affordances that are not feasible for average users.

^2^Not to mention COVID-19 constraints on in-person lab spaces.

^3^Moser and O'Keefe were jointly awarded the 2014 Nobel Prize in Medicine/Physiology for their landmark discoveries of grid and place cells.

^4^In robotics, the concept of dead reckoning describes the calculation of positionality of a moving object based on previously determined positions (Borenstein and Feng, 1994).

^5^In fact, microscopic videos of moving single cell organisms may also be attributed with human intentions and goals in line with the original Heider-Simmel simulation.

^6^HTC refers to this area as the “play area”, whereas Oculus refers to this area as the “guardian boundary”.

^7^In addition to the teleport function, there are other methods of seamless locomotion, such as redirected walking (Razzaque et al., 2005), which locks the rotation of the virtual world by holding down the button so that users may maximize their limited physical space to be used as an infinite play space.

^8^If however, movements are slow and incremental, movements and images may be processed every 10 frames as opposed to for each frame.

^9^Face-rigged avatars are primarily used by Virtual YouTubers (Zhao et al., 2019; Lu et al., 2021), such as Kizuna AI, although newer social media platforms such as ItsMe (https://www.itsme.video/) connect face-rigging avatars with social media functionality.

^10^ www.mixamo.com
